# Punishment on Pause: Preliminary Evidence That Mindfulness Training Modifies Neural Responses in a Reactive Aggression Task

**DOI:** 10.3389/fnbeh.2021.689373

**Published:** 2021-07-23

**Authors:** Hadley Rahrig, James M. Bjork, Camila Tirado, David S. Chester, J. David Creswell, Emily K. Lindsay, Jennifer Kim Penberthy, Kirk Warren Brown

**Affiliations:** ^1^Department of Psychology, Virginia Commonwealth University, Richmond, VA, United States; ^2^Department of Psychiatry, Virginia Commonwealth University, Richmond, VA, United States; ^3^Department of Psychology, Carnegie Mellon University, Pittsburgh, PA, United States; ^4^Department of Psychology, University of Pittsburgh, Pittsburgh, PA, United States; ^5^Department of Psychiatry and Neurobehavioral Sciences, University of Virginia School of Medicine, Charlottesville, VA, United States

**Keywords:** mindfulness, aggression, provocation, neuroimaging, emotion regulation, retaliation, punishment

## Abstract

Reactive aggression, a hostile retaliatory response to perceived threat, has been attributed to failures in emotion regulation. Interventions for reactive aggression have largely focused on cognitive control training, which target top-down emotion regulation mechanisms to inhibit aggressive impulses. Recent theory suggests that mindfulness training (MT) improves emotion regulation *via* both top-down and bottom-up neural mechanisms and has thus been proposed as an alternative treatment for aggression. Using this framework, the current pilot study examined how MT impacts functional brain physiology in the regulation of reactive aggression. Participants were randomly assigned to 2 weeks of MT (*n* = 11) or structurally equivalent active coping training (CT) that emphasizes cognitive control (*n* = 12). Following training, participants underwent functional magnetic resonance imaging (fMRI) during a retaliatory aggression task, a 16-trial game in which participants could respond to provocation by choosing whether or not to retaliate in the next round. Training groups did not differ in levels of aggression displayed. However, participants assigned to MT exhibited enhanced ventromedial prefrontal cortex (vmPFC) recruitment during punishment events (i.e., the aversive consequence of losing) relative to those receiving active CT. Conversely, the active coping group demonstrated greater dorsomedial prefrontal cortex (dmPFC) activation when deciding how much to retaliate, in line with a bolstered top-down behavior monitoring function. The findings suggest that mindfulness and cognitive control training may regulate aggression *via* different neural circuits and at different temporal stages of the provocation-aggression cycle.

**Trial Registration:** identification no. NCT03485807.

## Introduction

Aggression, the intention to harm someone against their will, is a serious public health concern. Behavioral treatments have not been universally effective, so it is critical that researchers investigate alternative interventions for preventing or reducing aggressive behavior (Fix and Fix, 2013; Bertsch et al., 2020). Aggression has been typologized into reactive [impulsive and hostile retaliation to perceived threat (Crick and Dodge, 1996)] and instrumental (primarily goal-driven) subtypes, each with distinct neurocircuit underpinnings and cognitive contributors that may in turn require different therapeutic approaches (Blair, 2001). Among evidence-based treatments, mindfulness-based interventions show potential for effectively reducing reactive aggression given that mindfulness practice has been linked to changes in neural function within executive control networks, associated with the inhibition of aggressive impulses (Bertsch et al., 2020), and emotion reactivity networks implicated in responsivity to emotional provocation (Hölzel et al., 2011; Tang et al., 2015). However, to date no studies have investigated the impact of mindfulness training (MT) on behavioral and neural indices of reactive aggression.

### Mindfulness Training to Reduce Reactive Aggression

Meta-analyses indicate that anger is a fundamental predictor of violence (Chereji et al., 2012; Birkley and Eckhardt, 2015); thus, violent offenders are frequently referred for anger management. Prevailing research on standardized interventions for anger and aggression has largely focused on the family of cognitive-behavioral therapies (CBT) (Lee and DiGiuseppe, 2018), particularly for the management of aggression in children and adolescents (e.g., Hoogsteder et al., 2015). Although literature concerning the use of aggression interventions for adults is relatively sparse (Lee and DiGiuseppe, 2018), meta-analyses of such studies indicate that the most effective programs integrate multiple therapeutic modalities (e.g., psychoeducation, cognitive training, family counseling) (Lee and DiGiuseppe, 2018). The majority of therapeutic packages rely on cognitive-behavioral approaches (Lee and DiGiuseppe, 2018), and so it is important to investigate additional therapeutic modalities that may be integrated into existing treatments. One promising adjuvant for the treatment of anger and aggression is MT, which centers on cultivating sustained, non-reactive attention to present moment experiences (Brown et al., 2015). Mindfulness has been theoretically positioned as an effective means to attenuate anger and aggression, given that training in non-reactance toward emotional experiences is commonly at the core of secular mindfulness interventions (Denson, 2015; Anālayo, 2018). Accordingly, an emerging body of research has begun to investigate the impact of mindfulness on aggression and associated mechanisms of action.

A wealth of research supports the effects of MT on cognitive and affective systems implicated in internalizing psychological conditions (e.g., depression; Keng et al., 2011; Lin et al., 2018), and early evidence suggests that such effects may generalize to externalizing behaviors as well, including aggression (Singh et al., 2007; Heppner et al., 2008; Borders et al., 2010; DeSteno et al., 2018; Gillions et al., 2019). Initial research broadly suggests that mindfulness may reduce aggression *via* improvements in emotion regulation (Gillions et al., 2019). However, there is little consensus regarding how mindfulness specifically interacts with the cognitive and affective mechanisms of aggression. According to the General Aggression Model (GAM) (Anderson and Bushman, 2002), aggression is the end product of a multi-stage process in which: (1) a trigger (i.e., provocation) elicits a combination of cognitive and affective reactions; (2) cognitive and affective factors influence appraisal of the situation; and then (3) these factors collectively inform the decision to aggress, either deliberately or impulsively. Neural frameworks of emotion regulation have been used to elaborate on this theory by mapping GAM stages onto distinct neural processes (Etkin et al., 2015; Fanning et al., 2017; Bertsch et al., 2020). Emotion regulation can manifest during early stages (e.g., reactivity to provocation) *via* modulation of regions involved in automated emotion reactivity (e.g., amygdala and insula) and valuation [e.g., ventromedial prefrontal cortex (vmPFC)], described as the automated encoding of subjective importance (Montague et al., 2006; Bertsch et al., 2020). Conversely, regulation at subsequent stages (e.g., appraisal and decision-making) recruits higher level cortical regions to deliberately re-evaluate the emotion trajectory and (re)consider actions. Acknowledging this theoretical framework, it remains unknown how mindfulness-based interventions alter neural processes of aggression management as it unfolds in real time.

This aggression model offers two temporal points for intervention to prevent an aggressive response to provocation, each with potentially different neuroanatomical substrates. First, cognitive training may promote the inhibition of aggression by improving capacity to reappraise provoking stimuli as a means to mitigate emotional impact. Such cognitive strategies implicate “top-down” neural circuitry in which prefrontal cortical engagement supports deliberate choice selection (Dosenbach et al., 2008). Conversely, cognitive training could downregulate the initial emotional impact of provocation by tamping down “bottom-up” processes that operate at relatively short time scales (Ochsner et al., 2009). Although several cognitive therapies may affect longer-scale “top-down” evaluative processes for retaliation, unique to MT, however, is the goal to disrupt initial reactivity to internal stimuli (e.g., thoughts and feelings) and environmental stimuli (e.g., others’ behavior) before cognitive change strategies like reappraisal are required (Hölzel et al., 2011; Tang et al., 2015; Guendelman et al., 2017). Although mindfulness and cognitive training programs have been similarly linked to improvements in top-down regulation (e.g., Zeidan et al., 2010; Buhle et al., 2014; Garland et al., 2015; Sevinc et al., 2019), mindfulness exclusively has been shown to target bottom-up pathways.

By practicing mindfulness in the context of emotional provocation, individuals learn to attend to unpleasant sensations without elaboration or judgment. Evidence suggests that with time, this practice can increase tolerance of difficult emotions, thereby reducing the need to consciously regulate or terminate emotional experiences in order to control them (e.g., Hölzel et al., 2011; Tang et al., 2015; Sevinc et al., 2019). Thus, it is plausible that MT alters bottom-up emotional reactivity to provocation, thus circumventing the use of emotion regulation strategies like reappraisal at later stages. This perspective is bolstered by research showing mindfulness to reduce physiological indicators of anger (e.g., respiration rate, heart rate, blood pressure) (Fennell et al., 2016) and anger rumination (Borders et al., 2010; Long and Christian, 2015) in the face of provocation. Despite these initial findings, stronger conclusions necessitate experimental approaches designed to isolate top-down from bottom-up processes theoretically implicated in the mindful regulation of aggression.

In a recent well-controlled study, DeSteno et al. (2018) examined whether MT could reduce aggression absent improvements in executive functioning. Participants randomly assigned to receive either 3 weeks of training in mindfulness meditation or daily logic assignments completed a lab-based assessment of aggression, during which participants were provoked by a stranger and then given the opportunity to retaliate aggressively. Relative to the control condition, individuals assigned to MT demonstrated significantly less aggressive behavior following provocation. The mindfulness and active control participants exhibited no differences on measures of executive control, suggesting that mindfulness may disrupt the initial generation of aggressive urges, thus circumventing the need to deliberately inhibit aggressive behavior through executive control mechanisms. Moreover, these findings support the position that the mindful regulation of aggression does not necessitate top-down regulation; to the contrary, implicit or bottom-up regulation may be sufficient. We sought to extend this research by examining the neural targets of MT and their association with both bottom-up and top-down regulation of aggression.

### Modeling Aggression and Its Regulation Using fMRI

Functional magnetic resonance imaging (fMRI) offers a unique opportunity to probe brain mechanisms of mindfulness and other cognitive interventions. Neural mechanisms of aggression have been researched extensively, and neuroimaging methods may be leveraged to investigate the brain-based effects of MT in the context of reactive aggression. Previous neuroimaging research on reactive aggression has prominently featured the Taylor Aggression Paradigm (TAP; e.g., Krämer et al., 2007, 2011; Lotze et al., 2007; Beyer et al., 2014; Repple et al., 2017), a laboratory task with strong convergent, discriminant, and external validity (Giancola and Zeichner, 1995). Notably, TAP indices have also been associated with self-reported measures of physical aggression (Giancola and Parrott, 2008). During the TAP participants compete against an ostensible opponent (actually a computer program) in a reaction time task. If the participant wins a trial, their ostensible opponent receives an aversive noise blast, the intensity of which is chosen by the participant. If the participant loses, they receive a noise blast at a volume level selected by the ostensible opponent. Because the opponent’s behaviors are pre-programmed, multiple levels of provocation can be simulated. By incorporating the TAP task into fMRI designs, researchers are able to evaluate neural indicators associated with reactive, or provoked aggression. Notably, the TAP allows researchers to dissociate neural reactions associated with each trial’s decision phase, in which the participant chooses a level of noise to administer, from neural reactivity associated with responses to opponent provocation or delivery of punishment (i.e., noxious stimuli) to the participant.

Investigation of these distinct phases may plausibly be applied to the isolation of emotion regulation mechanisms, with neural activity from the decision phase and the outcome phase corresponding to top-down and bottom-up mechanisms respectively. However, very few studies to date have reported neurocircuit recruitment during the provocation or outcome phase independent of the decision phase but see Lotze et al. (2007); Gan et al. (2015); Wagels et al. (2019). This is a critical gap in that mechanisms of aggressive responding, namely emotion reactivity and decision making, relate to dissociable biological pathways (Weidler et al., 2019), and aggression interventions may be tailored to target distinct neural and behavioral trajectories. This distinction may be particularly relevant in the context of provocation, given that top-down strategies are cognitively demanding and challenging to deploy within the short timescales of high intensity emotions (Shafir et al., 2016) or during cognitive fatigue (Bertsch et al., 2020). Thus bottom-up emotion regulation, such as may be conferred by mindfulness, may provide an advantage by reducing emotional responses in aggressive contexts without requiring, and by extension, reducing demand for top-down regulatory control.

Although findings differ across studies, the decision phase of the TAP has commonly been associated with engagement of the dorsolateral PFC (dlPFC), ventrolateral PFC (vlPFC), and dorsomedial prefrontal cortex (dmPFC) (Fanning et al., 2017). Broadly implicated in cognitive control (i.e., the inhibition of retaliation) and mentalization, these cortical regions theoretically support top-down regulation strategies, including emotion control and response selection, to deliberately inhibit aggressive action (Buhle et al., 2014; Bertsch et al., 2020). Moreover, these regions exhibit top-down functional and anatomical projections to subcortical (e.g., amygdala and insula) and cortical regions (e.g., vmPFC) (Ghashghaei et al., 2007) associated with impulsive emotional responding (Fanning et al., 2017).

The accumulation of research suggests that higher level cortical regions facilitate the downregulation of anger reactivity thereby promoting behavioral control over aggressive impulses (Bertsch et al., 2020). However, -less is known about the influence of bottom-up processes, especially given that the majority of fMRI studies do not investigate the provocation or punishment phases (i.e., losing outcome phase) directly (Fanning et al., 2017). In an exception to this trend, Lotze et al. (2007) demonstrated that the punishment phase was associated with activation from the vmPFC, a region implicated in the regulation of threat response (Sladky et al., 2015; Blair, 2016) and safety-signaling (Eisenberger et al., 2011), defined as the implicit downregulation of distress to noxious stimuli. Collectively reports of neural engagement during the provocation and punishment phases suggest that implicit regulation *via* bottom-up processes is relevant for the emotion regulation of aggression. If MT indeed enhances bottom-up regulation of aggression, it follows that mindfulness will engage neural regions associated with implicit emotion regulation, particularly the vmPFC, during phases preceding the decision to aggress, namely, the provocation or punishment phases of the TAP.

### The Present Study

Emerging research highlights mindfulness as a promising alternative to downregulating reactive aggression. While neuroimaging evidence suggests that MT modifies neural networks relevant to emotional reactivity and regulation (e.g., Hölzel et al., 2011; Opialla et al., 2015; Kral et al., 2019), no research has examined mindfulness-related neural mechanisms of reactive aggression. To address this question, the present pilot study investigated neural processes implicated in the initiation of, and response to reactive aggression among participants who completed MT. Participants were randomly assigned to 2 weeks of smartphone-delivered MT or to a structurally equivalent coping training (CT), the latter providing an active, well-matched control condition (Lindsay et al., 2018a, b, 2019a, b). The CT program, designed to train skills in top-down regulation strategies (including reappraisal and reframing) is well-suited to the present investigation. While distinct from mindfulness-related strategies, cognitive-heavy strategies similarly target emotion-behavior trajectories, to intercept a progression from perceived provocation to retaliation. Thus, comparison of these closely matched programs allowed us to disambiguate their common and divergent effects. We theorized that in the context of aggression regulation, mindfulness and cognitive training would similarly improve top-down control, detected during the TAP’s decision phase. However, unlike cognitive-based regulation, the implicit regulation conferred by mindfulness would ostensibly disrupt initial reactivity to provocation and punishment, thereby reducing the need for later-stage, controlled regulation. Hence the present study had three aims.

The first aim was to investigate the effects of MT on neural indicators of top-down cognitive control characterized by enhanced activation from inhibitory network regions (e.g., dlPFC, vlPFC, and dmPFC) when deciding whether or not to retaliate (i.e., the decision phase). The second aim was to explore the effects of MT on bottom-up emotional reactivity to being aggressed against (i.e., provocation and punishment phases), as indicated by enhanced activation from regions implicated in emotional significance and motivational value (e.g., limbic structures, vmPFC). Finally, to build upon prior research examining MT effects on behavioral indexes of aggression (e.g., DeSteno et al., 2018), the third aim was to evaluate the effects of MT, relative to active control training, on a behavioral indicator of reactive aggression, operationalized as average levels of noise chosen on retaliatory trials of the TAP (high noise levels chosen by the ostensible opponent) and non-retaliatory trials (low noise levels chosen by ostensible opponent).

## Materials and Methods

### Participants

Participants were 23 healthy, meditation-naive adults recruited from the Richmond, Virginia area. Prospective participants were pre-screened using an internet-administered survey and were considered for inclusion if they spoke fluent English, demonstrated access to a data-enabled smartphone and, for other study purposes, reported greater than average levels of stress over the past month as operationalized by scores of ≥5 on the 4-item version of the Perceived Stress Scale (Cohen et al., 1983; Warttig et al., 2013). Participants were excluded if they met any of the following criteria: major uncorrected sensory impairments or cognitive deficits, pregnancy, left-hand dominance, diagnosis of medical or psychiatric illness within the last 3 months, hospitalization within the last 3 months, change in medication regimen within the last 8 weeks, self-reported current drug abuse, presence of MRI safety risks (e.g., ferromagnetic implants, body weight > 300 lbs.), or a history of head trauma or seizures. All participants provided written informed consent to take part in the study, which was approved by the Institutional Review Board of Virginia Commonwealth University and registered with clinicaltrials.gov (Identification No. NCT03485807). Of the 23 participants who completed training, two were lost to post-training assessment and one was excluded from analyses due to excess movement during fMRI acquisition of the TAP task. Demographic characteristics of the final sample are shown inTable 1. Preliminary analysis determined that mindfulness and CT groups did not significantly differ in terms of age, gender, race, marital status, income, or education, nor for treatment credibility/expectancy (all *ps* > .09).

**TABLE 1:** Baseline characteristics of mindfulness and active coping conditions.

	Mindfulness (*n* = 9)	Active coping (n = 11)	
	***M* (SD)**	***M* (SD)**	***P***
	
**Age**	33.22 (7.58)	35.09 (8.71)	0.62
	***n (%)***	***n (%)***	***P***
	
**Gender**			**0.28**
Male	2 (22%)	5 (45%)	
Female	7 (78%)	6 (55%)	
**Race/ethnicity**			0.22
White/Caucasian	6 (67%)	5 (45%)	
Black/African American	1 (11%)	4 (36%)	
Hispanic or Latino	1 (11%)	0 (0%)	
Asian Indian	1 (11%)	0 (0%)	
Other/Mixed Race	0 (0%)	2 (18%)	
**Marital status**			0.30
Married	2 (22%)	5 (45%)	
Divorced	0 (0%)	1 (9%)	
Never Married	7 (78%)	5 (45%)	
**Annual household income**			0.88
Less than $25,000	1 (11%)	1 (9%)	
$25,000-$39,000	3 (33%)	3 (27%)	
$40,000-$54,000	2 (22%)	3 (27%)	
$55,000-$69,000	1 (11%)	1 (9%)	
$85,000-$99,000	0 (0%)	1 (9%)	
$100,000-$114,000	0 (0%)	1 (9%)	
$130,000-$144,000	1 (11%)	0 (0%)	
$160,000 or more	1 (11%)	1 (9%)	
**Education**			0.41
Some college/no	1 (11%)	2 (18%)	
**Degree**			
Bachelor’s degree	1 (11%)	4 (36%)	
Post-graduate degree	6 (67%)	5 (45%)	
	***M* (SD)**	***M* (SD)**	***P***
	
**Anger and aggression**			
AMI I	2.84 (0.71)	2.50 (0.50)	0.23
RPAO	12 59 (3.71)	13 82 (3 59)	54

*AMU, Angry Mood Improvement Inventory score (Bushman et al., 2001); BPAQ, Buss-Perry Aggression Questionnaire composite score (Webster et al., 2014).*

### Procedure

After providing written informed consent in an initial laboratory visit, participants completed a Qualtrics software-housed battery of self-report measures and a baseline fMRI assessment during which they completed an anatomical scan, a resting state functional scan, and other tasks for study purposes outside the scope of this report. Following these assessments, participants were randomly assigned to receive one of two 14-lesson smartphone-based interventions (one lesson per day for 14 consecutive days; Lindsay et al., 2018a, b, 2019a, b) delivering either MT or active CT. Participants returned to the brain imaging facility within 1–5 days of completing the final lesson of their training program. There, participants underwent a 45-min fMRI session during which participants completed an anatomical scan, a resting state functional scan, and the TAP retaliatory aggression task. Following the brain imaging session, participants completed follow-up questionnaires before being debriefed and dismissed.

#### Intervention Programs

The present study used a two-arm intervention design previously used to establish the efficacy of brief remote-delivered MT for stress reduction (Lindsay et al., 2018a). In addition to reducing subjective and biological markers of stress reactivity (Lindsay et al., 2018a), this MT program has been shown to reduce negative affect (Lindsay et al., 2018b), increase positive affect (Lindsay et al., 2018b), and improve social connectedness (Lindsay et al., 2019b). Relative to the mindfulness program, the active coping control program has been shown to minimally reduce stress, improve negative (but not positive) affect, and increase social connectedness (Lindsay et al., 2018a, b, 2019b). Although the effects of these training programs on social and emotional wellbeing have been established, this study is the first to examine their impact on anger and aggression outcomes.

Participants assigned to MT received instruction in present-centered, receptive attention with a focus on developing equanimity toward ongoing experiences, while participants assigned to the control condition received instruction in cognitive reframing and reappraisal strategies as well as guided imagery and problem-solving. The interventions were structurally equivalent and delivered *via* audio recordings from the same instructor. Each daily lesson was 15–20 min in length and included daily brief homework assignments (3–10 min per day). Each lesson taught specific techniques through didactic explanation, guided practice, and brief daily assignments designed to integrate mindfulness and coping skills into day-to-day experiences [see Lindsay et al. (2018a) for full training protocol]. Research assistants monitored daily progress to ensure lesson compliance and participants were encouraged to text or call a study hotline to ask questions or resolve technical issues. Research assistants contacted participants by phone on days 3 and 9 of their intervention to address difficulties or training-specific questions and encourage participant adherence.

#### Taylor Aggression Paradigm

**FIGURE 1:**
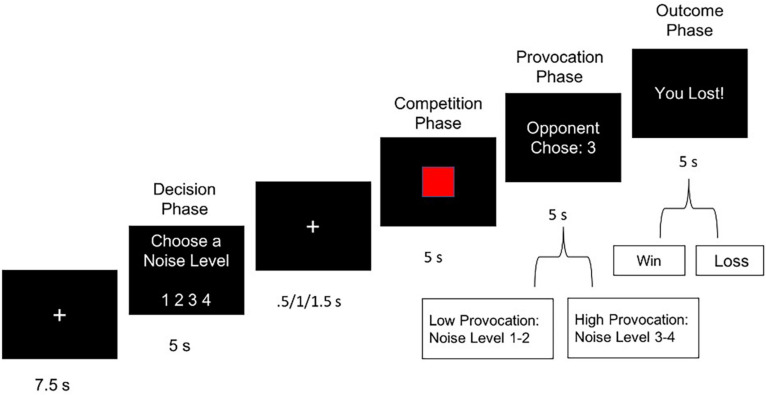
Single trial of the Taylor Aggression Paradigm (TAP), showing a loss outcome (i.e., punishment) with a high level of provocation.

To investigate the role of MT in altering neural signatures of retaliatory aggression, we administered a version of the TAP adapted for the fMRI scanner (e.g., Dambacher et al., 2015; Repple et al., 2017). Recent research supports flexible use of the TAP, as it has shown to be psychometrically robust to variations in sampling, laboratory settings, and analytical approaches (Hyatt et al., 2019; Lasko and Chester, 2021). Participants were informed that they would play an online computerized game with a participant situated in another lab. Participants were told that they would compete in multiple trials of a reaction time competition, in which the loser of each trial received an aversive noise blast through headphones, at one of four noise levels chosen by the other player. In reality, participants played against a preset computer program designed to produce four volume levels of white noise, with volume settings ranging from 1 (60 dB) to 4 (105 dB), in 22.5 dB intervals. The TAP consisted of 16 trials (Figure 1). Each trial began with a fixation phase, followed by a decision phase, in which participants selected the volume of noise blast that their partner would receive if their partner lost the reaction time trial. Participants then viewed a fixation cross with a jittered duration (0.5/1.0/1.5 s) before the competition phase, during which participants were required to quickly press a button when a red square target was shown on-screen (5 s). Participants then viewed their opponent’s (pre-programmed) volume setting. This time point of notification, when the participant perceived the opponent’s intended noise blast setting, was modeled as the provocation phase (see Figure 1). Finally, in the outcome phase, participants learned whether they won or lost the trial. The “losing” outcome phase, modeled as the punishment phase, subjected participants to a 5 s noise blast delivered by their opponent. Trials were characterized as retaliatory if they followed trials with high provocation (noise levels 3 or 4) and non-retaliatory if they followed trials with low provocation (levels 1 or 2). The 16-trial task contained eight retaliatory and eight non-retaliatory trials that were randomized across participants. Wins and losses were also randomly ordered across participants. Participants practiced the task first outside of the scanner to provide an opportunity for subjective evaluation of each noise level prior to neuroimaging assessment.

Following neuroimaging assessment, participants received self-report questionnaires assessing TAP-associated emotional reactivity and motivation to aggress. Participants completed the Aggressive Motives Scale (AMS) (Anderson et al., 2004), a 6-item scale measuring desire to harm their opponent during completion of the TAP. We also administered an adapted version of the Aggressive Pleasure Scale (APS) (Chester and DeWall, 2015), a 38-item self-report index of positive emotions (e.g., excited, proud) and negative emotions (e.g., distressed, ashamed) felt by the participant when their opponent received the noise blast.

#### MRI Acquisition and Preprocessing

Imaging was performed using a 3.0-T Phillips Ingenia MRI scanner. Blood oxygen level dependent (BOLD) signals were acquired using a T2^∗^-sensitive echoplanar sequence with a repetition time (TR) = 2,500 ms, echo time (TE) = 28 ms, flip angle = 90°, matrix size = 64 × 61, and field of view (FOV) = 224 mm. Each time series dataset contained a total of 213 volumes after removing the initial eight dummy volumes from analysis. Each functional volume contained 40 3.5-mm-thick parallel transverse slices. Structural scans were acquired using a T1-weighted MP-RAGE sequence (1 mm^3^ isotropic voxel size, TR = 2,500 ms, TE = 28 ms, flip angle = 90°, matrix size = 240 × 256, FOV = 240 mm) to facilitate registration to native space. Analysis of Functional NeuroImages (AFNI) software (Cox, 1996, 2012) was used to conduct all preprocessing and fMRI analyses. Individual time series datasets were despiked to compensate for motion artifacts, corrected for head motion (3dvolreg) with reference set to the middle volume, warped out to common stereotactic reference space (Montreal Neurological Institute; MNI), and spatially smoothed to uniform 6 mm full-width half maximum Gaussian kernel. Motion displacement reports were inspected in order to censor volumes exceeding ± 0.3 mm displacement in the *x*, *y*, or *z* directions.

### Statistical Analyses

#### Behavioral Data Analyses

Behavioral aggression was operationalized as participants’ noise volume selection (levels 1–4) during the decision phase of each trial. Volume selection was divided for analysis into retaliatory or non-retaliatory aggression trials, reflecting whether high or low noise levels, respectively, were chosen by the ostensible opponent on the immediately prior trial. To examine aggression across the 16 trials, multilevel models were constructed, which permitted control of within-person response variability across trials and, for exploratory purposes, examination of trajectories of response across trials. Analyses were conducted using SAS PROC MIXED (Wolfinger, 1997; Singer, 1998).

#### MRI Data Analyses

##### Whole-Brain Analyses

Functional neuroimaging data were analyzed using GLM as implemented by AFNI’s program, 3dDeconvolve (Cox, 1996, 2012). The multiple linear regression model included removal of mean, linear, and quadratic trends, and motion-related variance in the BOLD signal. Regressors for aggression, provocation, win trials, and loss trials were convolved with the gamma variate model (Cohen, 1997) of the hemodynamic response function. Linear contrasts were calculated to compare each condition against an implicit baseline [decision phase > baseline; provocation > baseline; outcome phase win > baseline; outcome phase lose (i.e., punishment) > baseline]. Cross presentation events and competition events were modeled as baseline parameters. Resulting contrast images were linearly registered to native space structural volumes before being spatially normalized to MNI stereotaxic space. Individual contrast volumes were submitted to a group-level, mixed-effects analysis using 3dMEMA (Cox, 1996, 2012). Clusterwise thresholding was implemented using second nearest neighbor clustering (3dClustSim) with a minimum cluster size of 20 voxels. Uncorrected *p* and FDR-corrected *q* were thresholded at 0.005 and 0.05 respectively.

##### Trial-by-Trial Parametric Analysis

The TAP applies an iterative or repeated-measures approach to operationalize reactive aggression (Chester, 2019). Such iterative measures maintain the advantage of modeling ecologically valid social encounters of aggression, in which two parties have multiple opportunities to retaliate, and potentially escalate to higher levels of aggressive responding. In this vein, multilevel modeling (MLM) approaches have been proposed as an additional or alternative approach to examine trajectories of neural responses across TAP trials (Chester, 2019). Thus, we conducted additional analyses using a multilevel modeling (MLM) framework to examine trajectories of neural activity (beta values) within regions showing significant group-level variation in whole-brain analyses.

A multivariate technique developed by Rissman et al. (2004) was adapted to model neural activation for every stage of every trial using separate covariates. First-level regression equations modeled each event of the 16-trial paradigm. Thus, 59 parameters of interest (5 baseline; 54 signal) were entered into the GLM corresponding to beta weights for 16 decision events, 16 provocation events, eight losing events, and eight winning events. The resulting parameter estimates (beta values) were sorted into their corresponding stages to form a beta series for each stage, with beta values representing estimated BOLD activity of each voxel relative to baseline. Beta series for each phase were extracted from voxels within 4 mm spherical regions of interest (ROIs), centered on peak activation voxels derived from significant clusters of the whole brain analysis.

Multilevel analyses of the beta series were conducted in SAS 9.4 PROC MIXED (SAS Institute, 2011). Multilevel modeling allows time serial data to be retained in its temporal form (in this case, the beta series). Thereby, within-subject variability is modeled rather than treated as error, the latter a consequence of aggregation. This modeling permits more strongly powered analyses, among other advantages, including the retention of participant data wherein missingness is found in the time series. Optimal variance-covariance structure (unstructured, variance components, Toeplitz, compound symmetry, or autoregressive) was determined through chi-square tests comparing the –2 restricted log likelihood model fit indices for each outcome. A compound symmetry variance-covariance structure was supported in both models reported below.

## Results

### Taylor Aggression Paradigm Behavioral Responses

Analyses first examined the effects of mindfulness (MT) vs. active CT on behavioral aggression. As anticipated from prior research (Fanning et al., 2017), MLM analyses showed there was a main effect of provocation level on behavioral aggression, such that high levels of provocation elicited higher retaliation on subsequent trials (MT *M* = 2.44, SD = 1.22; CT *M* = 2.03, SD = 0.77) than did low levels of provocation (MT *M* = 1.89, SD = 1.14; CT *M* = 1.91, SD = 0.79), *b* = −0.267, SE = 0.070, *p* = 0.001 (95% CI = −0.416, −0.122). There was no main effect of training condition on behavioral aggression, such that across both high provocation and low provocation trials, MT and CT participants did not differ in noise levels chosen [*b* = 0.126, SE = 0.436, *p* = 0.777 (95% CI = −0.791, 1.042)]. There was also no interaction between training condition and provocation level on levels of noise chosen [*b* = −0.042, SE = 0.142, *p* = 0.769 (95% CI = −0.320, 0.237)].

Post-session self-report questionnaires were used to examine associations between emotion-motivational processes and establish that the TAP paradigm elicited emotion-motivation processes, and that such processes affected behavioral aggression. Aggressive motives (AMS) scores (*M* = 2.15, SD = 1.06) were positively correlated with behavioral aggression during both high provocation, *r*(18) = 0.60, *p* = 0.001, and low provocation trials, *r*(18) = 0.651, *p* = 0.002. Similarly, evaluation of the Aggressive Pleasure Scale (APS) indicated that experiencing pleasurable emotions during win trials (*M* = 3.71, SD = 1.39) was positively correlated with behavioral aggression at both high [*r*(18) = 0.449, *p* = 0.047] and low levels of provocation [*r*(18) = 0.622, *p* = 0.003], respectively. Self-reported negative emotion during win trials was not significantly associated with any behavioral measures of aggression (*p* > 0.05). There was no effect of training on AMS scores [*t*(18) = 1.291, *p* = 0.213] or APS scores, for either positive or negative emotion reactivity, *t*(18) = 1.083, *p* = 0.293 and *t*(18) = −0.856, *p* = 0.40 respectively.

### Taylor Aggression Paradigm Whole-Brain Responses

Significant activation clusters elicited by each phase of the TAP are reported in Table 2 according to phase of the task. Of particular interest to this study were condition differences during the decision phase and the losing outcome (i.e., punishment) phase.

**TABLE 2:** FMRI BOLD responses across four phases of the Taylor Aggression Paradigm (TAP).

TAP phase		Brain region	Peak *x, y, z* (MNI)	Beta coefficient
Decision	CT > MT	Dorsomedial PFC	3, 30, 39	0.11
Provocation	MT > CT	Inferior temporal gyrus	44, −9, −45	0.50
	CT > MT	Cerebellum	0, −69, −3	0.18
Losing outcome	MT > CT	Inferior temporal gyrus	−45, 11, −45	0.67
		Ventromedial PFC	8, 39, −2	0.17
Winning outcome	–	–	–	–

*MT, mindfulness training; CT, coping training active control; *x, y, z*, MNI coordinates at peak blood level-dependent signal intensity voxel; PFC, prefrontal cortex. Minimum cluster threshold = 20 voxels; cluster FDR *q* < 0.05.*

#### Decision Phase

**FIGURE 2:**
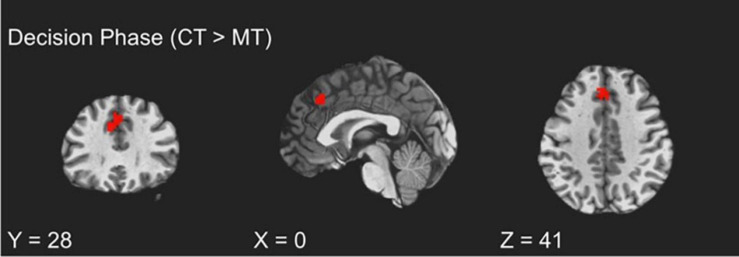
fMRI BOLD responses during decision to aggress with coping training condition exhibiting greater dmPFC activation relative to the mindfulness training condition.

To examine how BOLD activity during the TAP decision phase varied as a function of training assignment (MT vs. CT), linear contrasts for this phase were submitted to a whole-brain groupwise regression. The MT and CT groups exhibited significant differences during the decision phase, such that relative to MT, CT (CT > MT) participants exhibited greater recruitment from the dmPFC region (peak *t* = 3.65, *k* = 54; FDR-corrected *q* < 0.05, uncorrected *p* < 0.005, β = 0.11, peak MNI = 0, 28, 41; see Figure 2). Whole-brain analysis of the decision phase revealed no significant effect of training condition on bilateral dlPFC or vlPFC activity, FDR-corrected *q* > 0.05.

#### Outcome Phase

**FIGURE 3:**
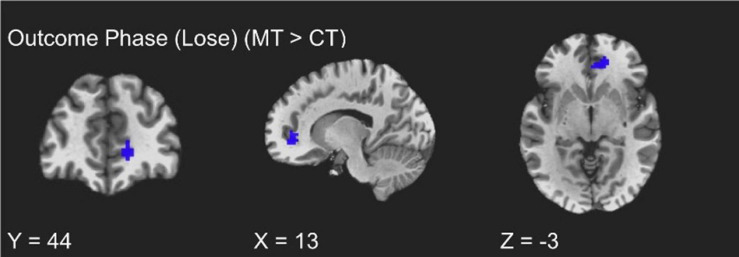
fMRI BOLD responses corresponding to loss trials of the outcome phase, with the mindfulness training condition exhibiting greater right vmPFC activity relative to the coping training condition.

The training groups did not show differing activations during the win trials of the outcome phase (uncorrected *p* > 0.01). However, assessment of condition differences within loss trials of the outcome phase (i.e., punishment events) revealed significantly greater activity from MT participants relative to CT participants localized to the right vmPFC (peak *t* = 3.70, *k* = 20; FDR-corrected *q* < 0.05, uncorrected *p* < 0.005, β = 0.17, peak MNI = 13, 44, −3; see Figure 3). Additionally, and unexpectedly, as Table 2 shows, punishment events were also characterized by group differences localized to the left inferior temporal gyrus (ITG), such that MT participants exhibited greater ITG activation relative to CT participants (peak *t* = 3.73, *k* = 20; FDR-corrected *q* < 0.05, uncorrected *p* < 0.005, β = 0.67, peak MNI = −45, 11, −45).

#### Provocation Phase

Table 2 also shows that training condition differences in ITG activation were found in the provocation phase of the TAP trials. Whole-brain contrasts indicated that MT participants exhibited greater right ITG activation relative to CT participants (peak *t* = 4.31, FDR-corrected *q* < 0.05, uncorrected *p* < 0.001, β = 0.50, peak MNI = 44, −9, −45). Group contrasts also revealed a significant cluster localized to the cerebellum, such that CT participants exhibited greater engagement relative to MT participants (peak *t* = 3.81, FDR-corrected *q* < 0.05, uncorrected *p* < 0.005, β = 0.18, peak MNI = 0, −69, −3).

### Complementary ROI Analyses

**FIGURE 4:**
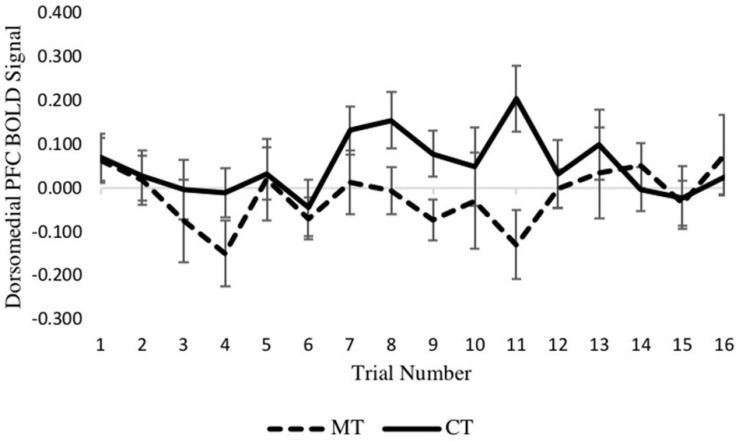
Between-condition BOLD activation within the dmPFC varies across the decision phase of each trial.

To confirm the main results regarding training condition differences in dmPFC and vmPFC, and to determine if neural activation varied across trials of the TAP, we constructed two multilevel models (MLMs) to examine decision phase and outcome phase beta series from voxels within ROIs derived from significant clusters of the whole-brain analysis. Using the decision phase beta series, a preliminary model that included main effects of retaliation, training condition, and trial number, and retaliation × training, and trial number × training interaction terms showed no interaction between retaliation and training condition (*p* = 0.643). Nor was an interaction between trial number and training condition found (*p* = 0.700). Thus these interaction terms were removed for the main models, which included only the main effects indicated above. As with the whole-brain analyses, MLM indicated a significant main effect of training type on left dmPFC activity (−6, 30, 38) during the decision phase [*b* = 0.070, SE = 0.021, *p* = 0.004, (95% CI = 0.026, 0.114)], with the CT group exhibiting significantly greater activity relative to the MT group. Examination of the 16 individual trials revealed that this condition effect was largely driven by trials in the middle of the TAP task (see Figure 4). However, there was no significant main effect of trial number (*p* = 0.515) nor retaliation trial type (*p* = 0.968).

**FIGURE 5:**
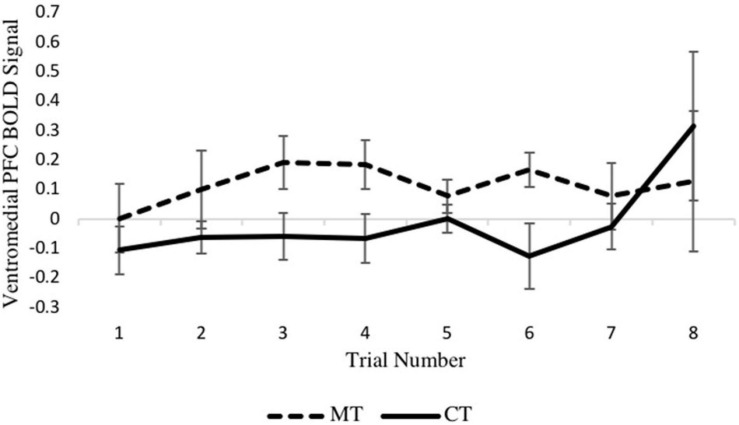
Between-condition BOLD activation within the right vmPFC varies across the loss outcome phase of each trial.

Turning to the second MLM analysis, concerning right vmPFC activity during the outcome phase, a preliminary MLM on loss trials that included main effects of training condition, trial number, and their interaction showed no training condition × trial interaction (*p* = 0.600) so was not further considered. In the main model, which included training condition and trial number as predictors, a main effect of training condition on right vmPFC activity was found [*b* = −0.132, SE = 0.056, *p* = 0.028, (95% CI = −0.249, −0.016)], with the MT group exhibiting significantly greater activity relative to the CT group. Examination of the eight individual loss trials (Figure 5) revealed that the beta series for each condition was mostly stable across trials of the task, and no main effect of trial was found (*p* = 0.462). Together, these MLM results support the whole-brain analyses reported earlier.

## Discussion

Aggression arises from heterogeneous sources of emotion dysregulation (Roberton et al., 2012; Bertsch et al., 2020); thus, it is critical to understand how different interventions uniquely target cognitive and affective systems implicated in aggression. Results of the present study suggest that MT, relative to training in cognitive coping skills, may target unique neural trajectories in the regulation of retaliatory aggression. As anticipated, condition differences were found in both the losing phase of the TAP as well as the decision phase, associated with bottom-up regulation and top-down regulation, respectively. Although groups did not significantly differ in terms of behavioral aggression, these results lend support for neural substrates theoretically involved in different interventions for retaliatory aggression.

### Decision Phase

Participants in the CT condition, relative to the MT group, exhibited greater recruitment from the dmPFC during the decision phase of the TAP. This finding was examined more closely using trial-by-trial parametric analysis (Rissman et al., 2004), which revealed that participants assigned to CT exhibited left dmPFC activity predominantly during middle trials of the task. The dmPFC is among prefrontal cortical structures that have previously been associated with domain-general cognitive control processes (Ochsner and Gross, 2008; Bertsch et al., 2020), and more specifically with the control of impulsive aggression (Fanning et al., 2017). Consistent with the findings presented here, prior research suggests that cognitive reappraisal – a skill central to the CT program – enhances recruitment from cognitive control regions, including the dmPFC (Buhle et al., 2014). In addition to its association with cognitive control, the dmPFC theoretically supports modulation of semantic representations (i.e., reappraisal of emotional stimuli) *via* connections to the lateral temporal cortex (Buhle et al., 2014). Hence, it is plausible that those trained in CT demonstrated increased capacity to alter representations of emotional stimuli (i.e., noise provocation) when deciding whether or not to retaliate.

Although the dlPFC and vlPFC are similarly implicated in cognitive control of aggression (Fanning et al., 2017), activation from these regions did not significantly differ between the CT and MT groups. These null results may be the product of overlapping mechanistic effects of CT and MT treatments. Previous research has shown MT to enhance activity in lateralized PFC regions (Farb et al., 2007, 2010) ostensibly associated with meta-cognitive skills (Chiesa et al., 2013; Kummar, 2018) which, while mechanistically unique to reappraisal skills, may similarly engage systems implicated in mindfulness. However, such conclusions necessitate further experimental research.

### Provocation and Punishment Phases

Results specific to the loss outcome (i.e., punishment) phase indicated significantly greater vmPFC engagement from participants assigned to MT relative to those assigned to CT. Although the vmPFC supports multiple functions including valuation, decision-making, and social cognition (Hiser and Koenigs, 2018), its role in the regulation of aggression may potentially be informed by the nature of social punishment modeled in the loss outcome trials Recent research indicates that the vmPFC is likely implicated in multiple mechanistic pathways of reactive aggression (Fanning et al., 2017), specifically threat reactivity and frustration (Bertsch et al., 2020). The involvement of the vmPFC in threat-related neurocircuitry is well-documented in both animal and human models. For example, research in rodents suggests that the vmPFC can implicitly downregulate threat responses (i.e., suppressing conditioned fear responses) *via* inhibition of the amygdala (Morgan et al., 1993), and human fMRI studies similarly implicate vmPFC neurocircuitry in the regulation of fear (Phelps et al., 2004; Blair, 2016) and enhancement of learned safety (Eisenberger et al., 2011). Alternative models, positioning the vmPFC within reward neurocircuitry (e.g., Buckner et al., 2008; Grabenhorst and Rolls, 2011; Levy and Glimcher, 2012; Bartra et al., 2013), suggest that vmPFC engagement may mediate the cost-benefit analysis needed to disengage from a retaliatory response (Blair, 2016; Bertsch et al., 2020). In this respect, the vmPFC may reflect awareness of the implications of retaliatory aggression, thereby enabling adaptive and flexible action.

The effect of MT on vmPFC engagement during loss trials is consistent with prior models of mindfulness-based emotion regulation. Mindfulness meditation has been shown to reduce habitual emotion reactivity (e.g., Safran and Segal, 1990; Bernstein et al., 2015, 2019; King and Fresco, 2019), and neuroscientific models posit that such effects are linked to fear extinction processes, facilitated through functional changes within the vmPFC (Hölzel et al., 2011; Kummar, 2018). In the context of social punishment, it is plausible that MT enhances vmPFC engagement in the face of a threatening stimulus (here, an aversive noise blast delivered by a competitor) and consequently reestablishes its emotional value or significance. While less is known regarding the impact of mindfulness on frustration and its mechanisms, MT has been shown to target neural networks relevant to conflict monitoring (Brewer et al., 2011). Functional changes to such networks, inclusive of the vmPFC, may potentially reduce retaliatory responses to provocation.

Notably, condition differences observed during the loss outcome trials extended to the inferior temporal gyrus (ITG), with the MT group exhibiting greater activation compared to the CT group. Moreover, this effect was also present during the provocation phase of the TAP, when participants viewed the level of noise selected by their opponent. Although we did not anticipate that MT would impact ITG activity during any phase of the TAP, this effect potentially informs our explanatory framework. The temporal pole is broadly implicated in the integration of visual and auditory stimuli and exhibits strong anatomical and functional connectivity with limbic regions and the vmPFC (Olson et al., 2007). Theoretical frameworks suggest that this functional circuitry may serve to process perceptual information and encode it as (non-semantic) social knowledge (Olson et al., 2013). Thus, it is possible that provocation-elicited ITG activation – operating in concert with the vmPFC – may underlie formation of emotionally laden social knowledge used to inform action. Such an explanation suggests that MT may enhance non-semantic (i.e., embodied) learning as a strategy to regulate aggression.

More speculatively, the heightened ITG activity observed here may reflect empathic concern in the context of the provocation-aggression cycle. This point receives some support from research with neurological patients, in which cognitive and affective empathy were critically impaired in those with temporal lobe damage, including but not limited to the ITG (Toller et al., 2015). Other work using a theory of mind and empathy task in neurotypical adults found overlapping activation in the ITG, among other regions (Völlm et al., 2006). Previous research has found higher ITG activation during meditation (Lazar et al., 2000; Hölzel et al., 2007; Kral et al., 2019) and higher gray matter concentration in this region in meditators (Hölzel et al., 2008). However, more research is needed to elucidate both the socio-emotional functions of the ITG and its potential functional and structural plasticity through mindfulness or other forms of mental training.

### Behavioral Findings

Regarding the behavioral results, levels of noise selected during the TAP did not differ between participants assigned to MT vs. CT, where participants from both training conditions responded with significantly higher levels of aggression when their opponent selected high levels of aggression. These findings suggest that the experiment successfully elicited aggression, and consequently, the regulation of aggression. Although we cannot infer that emotion regulation took place from the neuroimaging data, the neural effects reported in this study are consistent with those elicited during the regulation of aggression (Fanning et al., 2017). Additionally, we note that the similarity in rates of aggressive responding (intensity) between the two groups affords a critical interpretive advantage in that group activation differences were not likely driven by systematic groupwise differences in either schadenfreude (hedonistic pleasure from retaliation) or by systematic group differences in anticipation and apprehension of post-aggression escalation by the fictitious opponent.

It is also possible that the present findings are a consequence of methodological limitations associated with the TAP, particularly the version adapted to the fMRI context. Specifically, normative levels of aggression on the 4-level version of the TAP are subject to floor effects, given that healthy participants commonly restrict their responses to the lower volume levels (e.g., Krämer et al., 2007; Chester and DeWall, 2015; Chester et al., 2018). The behavioral responses reported here appear to illustrate such consequences of range restriction, as mean scores were uniformly low in both groups. Future research using more sensitive measures (i.e., permitting a greater range of response) may be better able to detect treatment-related effects if indeed present.

### Limitations and Future Directions

The conclusions drawn from this preliminary study are most notably limited by the small sample size, which reduced the power of the analyses to detect training condition effects. This study was funded by a modest grant, which placed constraints on the number of enrolled participants, who were scanned at pre- and post-training. Nevertheless, the present results inform candidate mechanisms concerning reactive aggression and mindfulness to help guide future research with well-powered samples. A second limitation concerns the cross-sectional design in which neural and behavioral indices of aggression were measured exclusively at post-training neuroimaging assessments. The current study is among the first to examine intervention effects using the TAP task; thus, the impact of habituation or sensitization associated with this task is poorly understood. In order to reduce the influence of possible confounds, we opted against a repeated measure design while attempting to establish baseline group equality using self-reported measures of aggression. While the proof-of-concept design used here aimed to identify candidate neural and behavioral targets of MT, we acknowledge that longitudinal designs are essential for inferring causal inferences. We recommend that future studies expand upon this research using controlled, repeated-measure designs with samples adequately powered to detect effects of interest. Additionally, researchers may consider using a passive control condition to dismantle the unique and overlapping effects of training in mindfulness and active coping strategies as they pertain to aggression interventions.

### Conclusion

Treating aggressive tendencies holds significant import to society. However, research has yet to elucidate the mechanisms of prominent interventions for aggression, including MT. The present preliminary study is among the first to investigate mindfulness-related neural associations of reactive aggression using a standardized laboratory task. The findings provide initial evidence demonstrating that mindfulness and generalized cognitive training may regulate aggression *via* divergent neural circuits and temporal stages of the provocation-aggression cycle. The results extend emotion regulation models of mindfulness (Chambers et al., 2009; Chiesa et al., 2013; Opialla et al., 2015; Tang et al., 2015; Kummar, 2018) by suggesting candidate mechanisms implicated in the regulation of aggression impulses. In particular, this research illustrates how neuroimaging procedures may be used to disambiguate top-down and bottom-up processes, which future research may reveal to be key to downregulating aggressive retaliation. More research in this domain will be relevant for the treatment of reactive aggression, a behavior which is influenced by multiple neural systems implicated in executive and emotional functioning.

## Data Availability Statement

The original contributions presented in this study are publicly available. This data can be found here: https://identifiers.org/neurovault.collection:9957.

## Ethics Statement

The studies involving human participants were reviewed and approved by VCU Institutional Review Board. The patients/participants provided their written informed consent to participate in this study.

## Author Contributions

KWB and DC contributed to the conception and design of the study. HR wrote the first draft of the manuscript. HR and JB performed statistical analysis. CT and HR developed study tools and performed data collection and curation. EL and JDC developed the intervention tools. KWB, DC, and JP contributed to funding acquisition. All authors contributed to the article and approved the submitted version.

## Conflict of Interest

The authors declare that the research was conducted in the absence of any commercial or financial relationships that could be construed as a potential conflict of interest.

## Publisher’s Note

All claims expressed in this article are solely those of the authors and do not necessarily represent those of their affiliated organizations, or those of the publisher, the editors and the reviewers. Any product that may be evaluated in this article, or claim that may be made by its manufacturer, is not guaranteed or endorsed by the publisher.

## Abbreviations

dmPFC: dorsomedial prefrontal cortex
vmPFC: ventromedial prefrontal cortex.
